# Dataset of elemental compositions and pollution indices of soil and sediments: Nile River and delta -Egypt

**DOI:** 10.1016/j.dib.2019.105009

**Published:** 2019-12-24

**Authors:** Wael M. Badawy, Octavian G. Duliu, Marina V. Frontasyeva, Hussien El-Samman, Sergey V. Mamikhin

**Affiliations:** a Egyptian Atomic Energy Authority (EAEA), Nuclear Research Center, Radiation Protection & Civil Defense Dept, 13759, Abu Zaabal, Egypt; b University of Bucharest, Faculty of Physics, Department of Atomic and Nuclear Physics, 405, Atomistilor Str, 077125, Magurele, Romania; c Frank Laboratory of Neutron Physics, Joint Institute for Nuclear Research, 6, Joliot Curie Str. 141980, Dubna, Russian Federation; d Menoufia University, Faculty of Science, Department of Physics, Shibin El-koom, Egypt; e Faculty of Soil Science, Moscow State University, Moscow, 119991, Russia

**Keywords:** Nile and delta, Soil and sediments, Major and trace elements, INAA, Pollution indices

## Abstract

This data is the first comprehensive baseline data on the geochemical composition of soil and sediments along the Nile River and Delta in Egypt that was subjected and analyzed by instrumental neutron activation analysis INAA. These data supported the research articles that were done to evaluate the elemental compositions and pollution sources in 176 sampling locations through 133 soil and 43 sediments samples along the Egyptian section of the Nile River and Delta – Egypt. “Geochemistry of sediments and surface soils from the Nile delta and lower Nile valley studied by epithermal neutron activation analysis” Arafa [1], “Major and trace element distribution in soil and sediments from the Egyptian central Nile valley” Badawy [2], and “Assessment of industrial contamination of agricultural soil adjacent to Sadat city, Egypt” Badawy [3]. The samples were analyzed by means of instrumental neutron activation analysis INAA and the concentrations in mg/kg of 28 major and trace elements are obtained. The quality control of the analytical measurements was carried out using different certified reference materials. Multivariate statistical analyses were applied. A total of eight individual and complex pollution indices were calculated in terms of the quantification of pollution extent and selection of the proper index based on the method and purpose of calculations. The spatial distribution of pollution load index PLI was mapped using GIS-technology. The normalized concentrations of the determined elements show no significant difference between soil and sediments concentrations and this, however, may be explained by the fact that origin of soil mainly is the sediments. To a clear extent, the concentrations of Ti (8017, 9672 mg/kg), V (124, 143 mg/kg), Cr (126, 160 mg/kg), and Zr (296, 318 mg/kg) are observed to be high in soil and sediments, respectively relative to other elements. Zr/Sc ratio shows a reduced sedimentary recycling and this may be explained by the tremendous influence of Aswan High Dam in preventing sediments supply from Ethiopian Highlights. Eventually, the pollution indices prove their suitability for assessing the individual and integrative contamination and show that there is no overall contamination. However, there are some contaminated localities mainly in Delta and mostly due to the dense population and anthropogenic activities. The data can be used as a raw data for constructing the first ecological atlas and evaluation of the ecological situation in terms of geochemistry and pollution.

**Table:** Specifications Table

Subject	Environmental Science
Specific subject area	Utilization of nuclear and related analytical techniques in environmental studies. Instrumental neutron activation analysis INAA and gamma ray spectrometer were used to measure the concentrations mg/kg of elements in soil and sediments samples.
Type of data	Maps, Figures, Tables, in excel file (*.xlsx)
How data were acquired	After the field sampling, the soil and sediments samples were subjected to instrumental neutron activation analysis (INAA). The obtained spectra were accumulated by means of gamma-ray spectrometer. Hence the data was processed by a developed software to calculate the concentration of elements. Details on the used analytical technique and the implemented approach to the calculated pollution indices are given in the section of Experimental Design, Materials, and Methods.
Data format	Raw and analyzed data are provided in an excel file including four sheets (raw data) and the analyzed are pollution indices, Fig. 1SM, and Fig. 2SM (supplementary material)
Parameters for data collection	Field collection of soil and sediments along the Egyptian Section of the Nile River and Delta.
Description of data collection	A total of 133 soil and 43 sediments samples were collected from the two banks along the Nile River and Delta to cover almost all the dense populated areas. To leave no doubt that there was no contamination from the used instruments in the sampling process, we have used non-metal instruments. The locations were registered using GPS and the map is provided. Soil samples were collected at depths 17–45 cm. while sediment ones were collected from the surface on the two banks of Nile River at depths 1–3 m
Data source location	Nile River and Delta - Egypt (latitude 26.8205528, longitude 30.8024979)
Data accessibility	With the article

**Table:** 

**Value of the Data** • Knowledge of the elemental composition gives a better understanding about the geochemistry of soil and sediments of Nile River and Delta. For the first time in Egypt a comprehensive baseline data is given about the major and trace elements in agricultural soil and surface sediments along the Nile River. It can be used to distinguish between the natural content of elements and the anthropological concentrations • These data can be used as a supportive tool to the decision makers in all the regulatory bodies related to agricultural and industrial fields. Ministries of ecology, industry, and agriculture can use these data for more interpretation and explaining some issues. • These data can be considered as a background or a baseline for construction an ecological atlas for Egypt in terms of major and trace elements. It can be used to examine any dynamics or changes in the future.

## 1 Data description

**Fig. 1:**
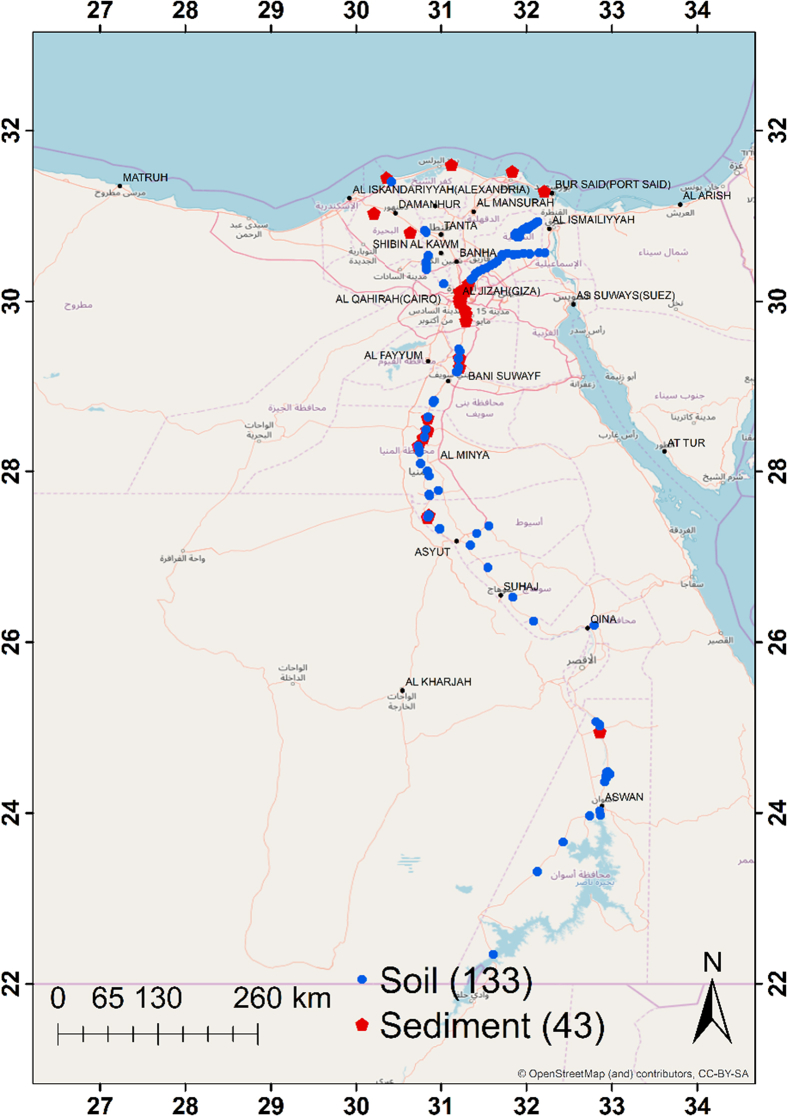
A map of the sampling localities.

Nile River is one of the longest rivers in the world and is the artery of fresh water for 11 Nile River basin countries [4,5]. Nowadays, many factors affect the sharp decrement of water quality and sedimentological processes, for instance industrial, domestic, and agricultural pollution. Since the construction of the High Dam in Aswan 1964, the flow of the Nile cycle and sediment discharge has been disrupted [6]. These data were extracted in the period from 2011 to 2017 by collecting 176 samples (133 soil and 43 sediments) along the two banks of the Egyptian Nile River and Delta as in Fig. 1. The elemental compositions in mg/kg of 28 major and trace elements (Na, Mg, Al, Ca, Sc, Ti, V, Cr, Mn, Fe, Ni, Co, Zn, As, Br, Rb, Sr, Zr, Sb, Ba, Cs, La, Ce, Sm, Tb, Hf, Th, and U).

**Fig. 2:**
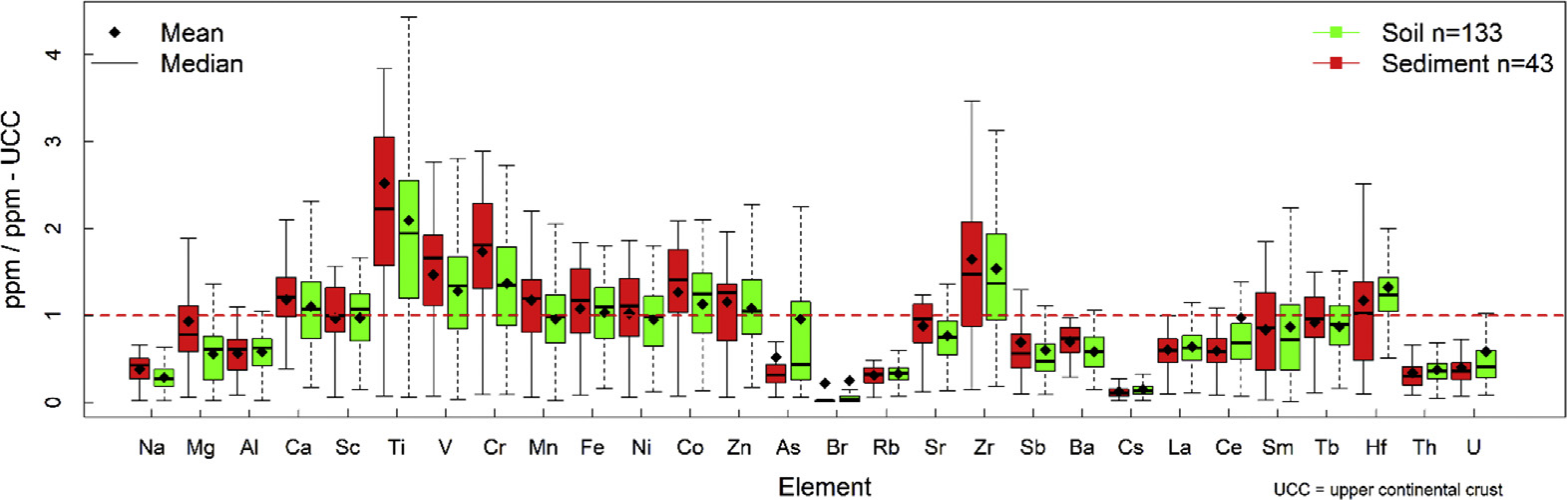
Boxplot illustrates the normalized concentration to the corresponding values of the upper continental crust UCC of 28 elements in soil and sediment samples.

**Fig. 3:**
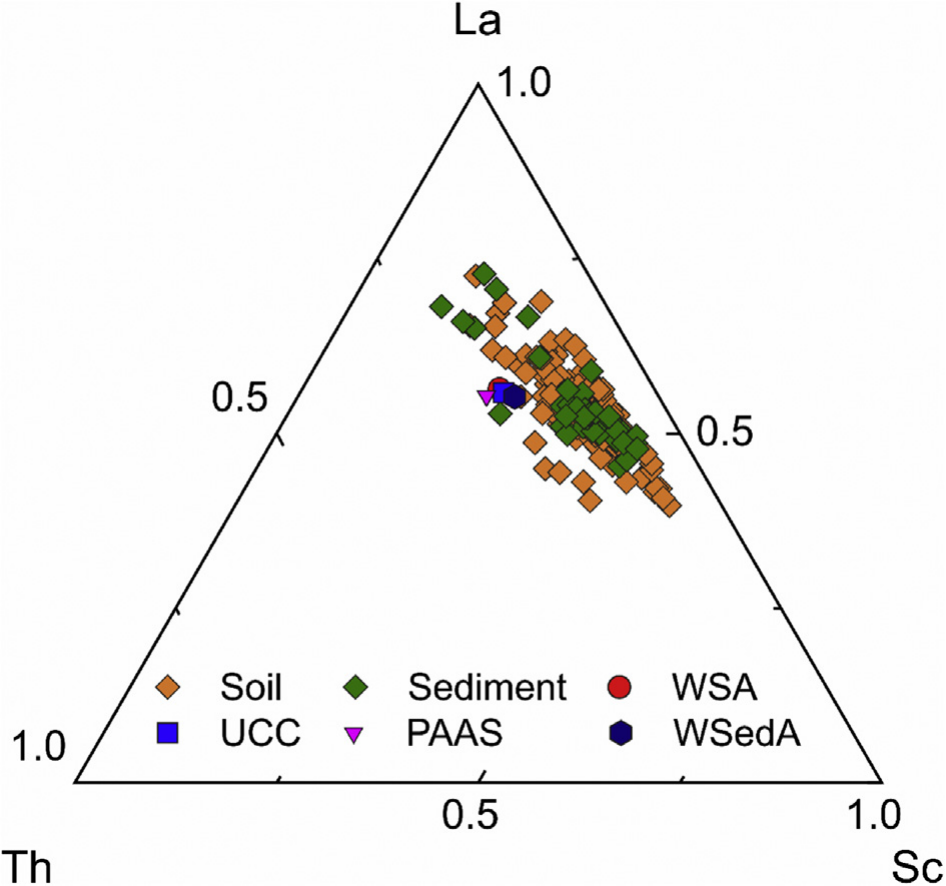
Ternary discriminating plot of Sc-La-Th, illustrates a good matching between the obtained data and those reported for upper continental crust UCC by Rudnick and Gao [7], for world average sediments WSedA by Viers [8], for Post-Archean Australian shale average PAAS by Taylor and McLennan [9], for world average soil WSA by Kabata-Pendias [10].

**Fig. 4:**
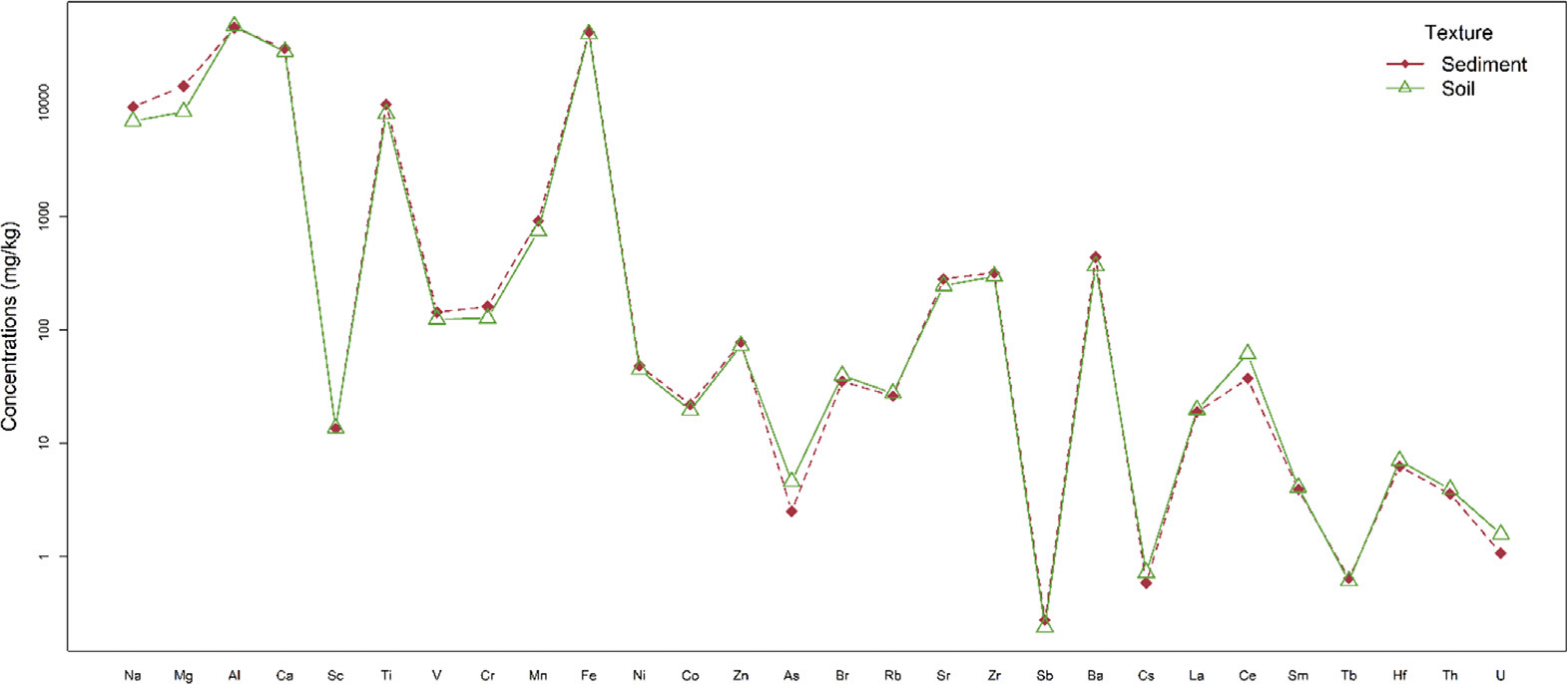
Interaction plot illustrates the difference of the mean values of soil and sediments for 28 elements.

The obtained data are provided in the supplementary materials Table SM1 and descriptive statistics are shown in Fig. 2. Furthermore, to elucidate the sources of pollution; eight pollution indices (6 complex and 2 individual) were calculated and their outcome was given in Table SM2. The description, features of the studied areas, discussion, and interpretations of findings are given in details in Arafa [1]; Badawy [2]; Badawy [3]. Multivariate statistical analysis was employed to extract more information about the provenance of soil and sediments as in Fig. 3. The discriminatory analysis shows broadly similar traits between soil and sediments. Both soil and sediments are in a good matching with the corresponding values reported for upper continental crust UCC by Rudnick and Gao [7], for world average sediments WSedA by Viers [8], for Post-Archean Australian shale average PAAS by Taylor and McLennan [9], for world average soil WSA by Kabata-Pendias [10]. The interaction plot as illustrated in Fig. 4 proves this finding, as the soil and sediments data are in line, except a slight difference in case of Na, Mg, Ti, V, As, and U.

**Fig. 5:**
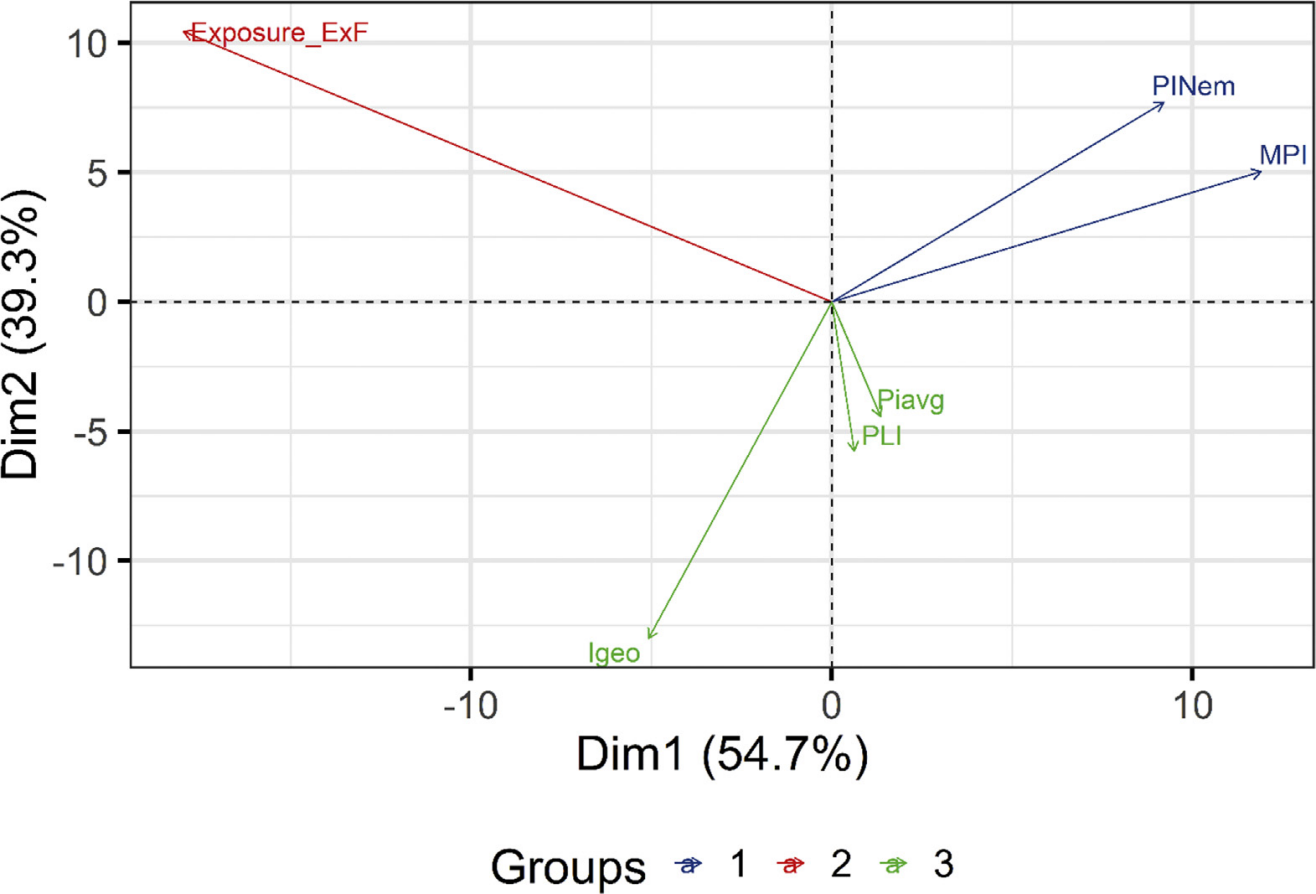
Principal component (PCA) analysis biplot of selected complex and individual indices.

Principal component analysis PCA and cluster analysis CA were used to group symmetrical geochemical elements and the highest contribution of soil and sediments to the 1st two PCAs (individuals and variables) is given in Fig. SM1. The pollution indices were calculated and PCA was used to get the proper pollution index as in Fig. 5. The spatial distribution of the pollution load index PLI is given in Fig. SM2.

## 2 Experimental design, materials, and methods

A total of 176 soil samples (133) and sediments (43), each weighing about 1 kg, were collected from the two banks along with the Egyptian sector of the river Nile and Delta as shown in Fig. 1. The samples were collected in conformity with the recommendations suggested by IAEA [11]. The soil samples were collected by the systematic grid sampling protocol (50 m × 50 m) from the accessible areas along the Nile River and Delta as well at 17–45 cm depth. Soil samples were collected from rural and urban areas. The soil texture was clay, sandy, and silty clay mixed sources, While the sediment samples were taken from the banks of the Nile River and floodplain at 1–3 m depth where the nearest point to the water level. The sediments mainly were silty clay and silty clay loam. The collected samples were twice pretreated; (i) the samples were thoroughly cleaned of plant debris, any other extraneous materials, and air-dried at room temperature to a constant weight. Later, they were grinded and homogenized using an agate ball mill. Finally, 100 g of each sample were zip-packed and sent to be subjected to epithermal neutron activation analysis at REGATA station at IBR-2M pulsed reactor in Frank Laboratory for Neutron Physics – Joint Institute for Nuclear Research – Dubna – Russian Federation. (ii) Around 0.1 g of each sample was wrapped in polyethylene and aluminum cups for short- and long-term irradiations, respectively. The samples were irradiated in channels equipped with the pneumatic system installed at REGATA station in the IBR-2 pulsed nuclear reactor of FLNP with the average power of two MW – Dubna – Russian Federation. The main characteristics of the irradiation channels are published by Frontasyeva and Pavlov [12]. However, a concise description of the analytical scheme for soil and sediments will be presented. To determine the short-lived isotopes in soil and sediment samples, each sample was irradiated for 1 min in channel 2, after 3–5 min of decay, was measured for 15 min. The distance between irradiation and measurement positions 60 m and the transportation time for polyethylene capsule is 10–20 sec. However, in case of determining the long-lived isotopes; samples were irradiated for approximately 3 days in the Cd-screened channel 1 with a neutron flux of 1.8 × 10^11^ n/cm^2^.sec. Samples were repacked and measured twice. The 1st time is after 7 days of decay for 45 min. While the 2nd time after approximately 30 days of decay for 90 min. In this case the distance is 70 m and the transportation time for aluminum capsule is 3–8 sec. Gamma spectra of the samples were measured by Ge (Li) detector or by HPGe detector with the resolution of 2.5–3 keV or of 1.9 keV, respectively, for the 1332 keV line of the ^60^Co. The software Genie 2000 was used to store, display, and analyze the gamma spectra. The other software developed at FLNP was used to calculate concentrations of the elements in the samples. The analytical errors of the concentrations of the elements of interest range from 3 to 15%. More details about irradiation time for short and long-lived isotopes, neutron flux, channels, pneumatic transport system of the REGATA installation, and automation system for measurement using sample changer were published elsewhere [[1], [2], [3],[12], [13], [14]].

The quality control of the analytical measurements using NAA was carried out using certified reference materials NIST standard reference materials (SRM) 1547- Peach Leaves, NIST SRM 1575а- Pine Needles, NIST SRM 1633b - Coal Fly Ash, NIST SRM1632с - Coal (Bituminous), NIST SRM 2709 – San Joaquin Soil, IRMM SRM 667 - Estuarine Sediment. SRM material varied between 1% and 10% with the exception of Rb, Ti, Ni, Mo, Au, Hf, W, and I for which the differences were 17% for Rb, 20% for Ti and Ni, 30% for Mo and Au, 33% for Hf and W, and 39% for I.

In order to calculate the pollution indices, the assessment of the geochemical background should be provided. Having a background value or a baseline value of the element in the examined soil or sediment samples is useful in terms of distinguishing between the natural content of elements and the anthropological concentrations. Therefore, two kinds of background were reported by Kowalska [15]. Reference and local or natural geochemistry background. The average content of heavy metals given in the literature, which can vary greatly due to localization differences and soil type, could be considered the reference geochemical background RGB. While the local or natural geochemical background LGB is the concentration of heavy metals conditioned by natural processes characteristic of a particular area [16,17]. In these data, the reference geochemical background of the upper continental crust UCC values reported by Rudnick and Gao [7] were considered. The pollution extent was quantified based on two approaches the individual and complex pollution indices. The individual levels of pollution from each analyzed metals can be calculated using individual pollution indices. While complex pollution indices describe contamination of soil in a more integrated approach, considering the content of more than one heavy metal or a sum of individual indices. The indices, used formula, parameters, description, and interpretation classes for the most widely used pollution indices based on different approaches are summarized in Table 1.

**Table 1:** Indices, used formula, parameters, description, and interpretation classes for the most widely used pollution indices based on different approaches.

#	Indices	Used formula	Parameters	Description	Interpretation
Individual pollution indices					
1	Enrichment factor EF	$EF=\;{\left(C_{x}/C_{Fe}\right)}_{sample}/{\left(C_{x}/C_{Fe}\right)}_{reference}$	(*C*_*x*_/*C*_*Fe*_)_*sample*_ is the ratio of the concentration of the element in the sample and the concentration of Fe in the sample while (*C*_*x*_/*C*_*Fe*_)_*reference*_ is the ratio of the same element in the worldwide average of UCC [7]	It is used to distinguish between the amplitudes of anthropogenic metal pollution relative to the background or reference elements	• EF < 1 No enrichment • 1 < EF > 3 Minor enrichment • 3 < EF > 5 Moderate enrichment • 5 < EF > 10 Moderate to strong enrichment • 10 < EF > 25 Strong enrichment • 25 < EF > 50 Very strong enrichment • EF > 5 Extremely strong enrichment
2	Geoaccumulation Index I_geo_	$I_{geo}=\;log_{2}\;\left(C_{n}/1.5\;B_{n}\right)$	C_n_ is the concentration of the element in the enriched samples, and the B_n_ is the background value of the element. Factor 1.5 is introduced to minimize the effect of possible variations in the background values, which may be attributed to natural lithological processes in soil and sediment [16,18].	It is widely used to assess the anthropogenic impact on soil and sediments. *I*_*geo*_ was proposed by Muller [19] to quantify the contamination of the metals above the background ones. The approach assesses the degree of metal pollution in terms of interpretation classes based on the increasing numerical values of the index.	• Igeo <0 Uncontaminated • 0 < Igeo <1 Uncontaminated/moderately contaminated • 1 < Igeo <2 Moderately contaminated • 2 < Igeo <3 Moderately/strongly contaminated • 3 < Igeo <4 Strongly contaminated • 4 < Igeo <5 Strongly/extremely contaminated • 5 < Igeo Extremely contaminated
3	Single Pollution Index PI	$PI=\;C_{n}/C_{B}$	C_n_ – the content of the element in soil and sediment, and C_B_ –values of the geochemical background	It determines which element has the highest impact for a soil and sediment environment	• PI < 1 absent • 1 < PI < 2 low • 2 < PI < 3 moderate • 3 < PI < 5 strong • PI > 5 very strong
Complex pollution indices					
4	Sum of contamination ∑PI	$\sum^{}PI=\;\overset{n}{\underset{i=1}{\sum }}PI$	PI – calculated values for Single Pollution Index and n – the number of total elements analyzed in each examined point	It gives the summing up of all PI for each element for each profile.	It mainly depends on the increasing numerical values and has interpretation classes. The higher the index, the higher the contamination for sampling profile.
5	Pollution Load Index PLI	$\text{PLI}=\;\sqrt[{n}]{\overset{n}{\underset{i=1}{\prod }}PI_{i}}$	n is the number of analyzed metals and PI is the calculated values for the single pollution index	It quantifies the degree of contamination in the entire sampling profiles. This index provides an easy way to prove the deterioration of the soil and sediments conditions because of the accumulation of metals.	When PLI >1, it means that pollution exists; otherwise, if PLI <1, there is no metal pollution
6	Average Single Pollution Index PI_avg_	$PI_{avg}=\;\frac{1}{n}\;\overset{n}{\underset{i=1}{\sum }}PI$	n denotes for the number of examined metals and PI is standing for the single pollution index.	It estimates the quality of soil and sediments, *PI*_*avg*_ was first employed by Qingjie [20]	*PI*_*avg*_ values higher than unity show a lower soil or sediment quality, which is conditioned by a high contamination level
7	Nemerow Pollution Index PINem	$PI_{Nem}=\;\sqrt{\frac{{\left(\frac{1}{n}\;\sum_{i=1}^{n\;\;}PI\right)}^{2}+\;PI_{max}^{2}}{2}}$	PI is the average calculated values for the single pollution index over the number of metals n, PImax is the maximum value of the pollution indices of all metals	It is applied to assess soil environmental quality. It is utilized for the degree of soil environmental pollution and integrative assessment of soil environmental quality and is given as follows [21,22]	• ≤ 0.7 Safety domain • 0.7–1 Precaution • 1–2 Slight • 2–3 Moderate • ≥ 3 Serious
8	Modified pollution index MPI	$MPI=\;\sqrt{\frac{{\left(\frac{1}{n}\;\sum_{i=1}^{n\;\;}EF\right)}^{2}+\;EF_{max}^{2}}{2}}$	EF is the average calculated values for the enrichment factor over the number of metals n, EF_max_ is the maximum value of the enrichment factor of all metals in the examined site [23]	It is used to eliminate the drawbacks and limitations that were found in other pollution indices and in particular, Nemerow Pollution Index PINem. The developed MPI has the same concept as PINem; however, it is based on the enrichment factor, not on the single pollution index PI. Both of MPI and PINem are used to the integrative assessment of soil environmental quality. the advantages of using MPI over PINem are i) consideration of a non-conservative behavior of sediments due to normalization in EF calculations, and ii) accurate thresholds for sediment qualification.	• MPI ≤1 Unpolluted polluted • 1 < MPI<2 Slightly polluted • 2 < MPI<3 Moderately polluted • 3 < MPI<5 Moderately-heavily polluted • 5 < MPI<10 Heavily polluted • 10 < MPI Severely polluted
9	Exposure factor ExF	$ExF=\;\sum^{}\frac{C_{n}-C_{av}}{C_{av}}$	C_n_ is given for the concentration of the metal in an analyzed sampling point, and C_av_ is the average concentration of metal in the soil and sediment samples	It is a helpful approach to mark where the highest metal loads in a given study site are located.	positive value denote for the existence of pollution, negative one refers to a metal depletion in the sampling profile, and value close to zero expresses about the background baseline.
